# Deciphering the Hantavirus Host Range Combining Virology and Species Distribution Models with an Emphasis on the Yellow Pygmy Rice Rat (*Oligoryzomys flavescens*)

**DOI:** 10.1155/2023/2730050

**Published:** 2023-04-19

**Authors:** Andrés Cabrera, David Romero, José Carlos Guerrero, Mario Clara, Adriana Delfraro

**Affiliations:** ^1^Sección Virología, Departamento de Biología Celular y Molecular, Instituto de Biología, Facultad de Ciencias, Universidad de la República, Montevideo, Uruguay; ^2^Grupo de Biogeografía, Diversidad y Conservación, Departamento de Biología Animal, Facultad de Ciencias, Universidad de Málaga, Málaga, Spain; ^3^Laboratorio de Desarrollo Sustentable y Gestión Ambiental del Territorio, Instituto de Ecología y Ciencias Ambientales, Facultad de Ciencias, Universidad de la República, Montevideo, Uruguay; ^4^Instituto de Ecología y Ciencias Ambientales, Facultad de Ciencias, Universidad de la República, Montevideo, Uruguay

## Abstract

Hantaviruses are the causative agents of hantavirus pulmonary syndrome (HPS) in the Americas. In Central and South America, 28 hantavirus lineages were associated with different Sigmodontinae rodents. Of these, Lechiguanas hantavirus was initially described as a lineage associated with HPS cases in the central region of Argentina. Initial studies on the rodent hosts and viral lineages performed between 1999 and 2005 showed that HPS cases in Uruguay were distributed mostly in the southern region of the country, and that the Lechiguanas hantavirus (LECV) and the closely related Andes Central Plata hantaviruses were the viral lineages most frequently associated with HPS cases, both carried by the yellow pygmy rice rat (*Oligoryzomys flavescens*). Although these rodents are present all across the Uruguayan territory, determining the extent of the risk areas for hantavirus transmission based on the distribution of the infected rodents may be a useful tool for disease control and prevention. Distribution models are positioned as an effective instrument in the prediction of diseases affecting human health. Assessment of the potential distribution of rodent reservoir hosts and analysis of the influence of environmental factors on hantavirus transmission can help to understand the spatial patterns of disease transmission risk. In the present study, virological studies and species distribution models were integrated to understand the hantavirus infection risk pattern in Uruguay. Virological analyses confirmed that in Uruguay, the primary hantavirus reservoir host for both viral lineages is the yellow pygmy rice rat. Additionally, we report an Azara's grass mouse (*Akodon azarae*) infected with the Andes Central Plata viral lineage. Based on the seropositive and nonseropositive yellow pygmy rice rats tested, the distribution models emphasized that favorable environmental conditions for the infected rodents are mainly related to the availability of human-disturbed rural environments with high humidity. We conclude that the innovative application of the methodologies reported herein allowed for the assessment of the current risk territory for HPS in Uruguay.

## 1. Introduction

Hantaviruses belong to the family Hantaviridae, order Bunyavirales. The Hantaviridae family has only one genus (*Orthohantavirus*) and 38 species recognized by the International Committee on Taxonomy of Viruses (ICTV) [1]. The viral particles are spherical, enveloped, have a diameter between 80 and 120 nm, and contain three negative single-stranded RNA genome segments designated small (*S*), medium (*M*), and large (*L*) [2]. Hantaviruses were first recognized as the causative agent of an epidemic of hemorrhagic fever in the troops fighting the Korean War in 1950 [3].

In the Americas, hantaviruses are the causative agents of a different disease, the hantavirus pulmonary syndrome (HPS). The association between hantaviruses and HPS was unveiled after an outbreak in the Navajo Reserve at Four Corners, Colorado, USA [4]. Further studies enabled the recognition of at least 10 hantavirus lineages indigenous to North America, hosted by Cricetidae rodents from the Arvicolinae, Sigmodontine, and Neotominae subfamilies. The first HPS outbreak in South America was reported in 1995 at El Bolsón, Río Negro province, Argentina, caused by a newly found hantavirus, the Andes virus, carried by the long-tailed pygmy rice rat (*Oligoryzomys longicaudatus*) [5]. Since then, at least 28 hantavirus lineages have been reported in Central and South America, associated with different Sigmodontinae rodents (reviewed in [6]). In 2018, infections with Araraquara (ARAV) hantavirus were described in two bat species; however, the role of these mammals as reservoir hosts deserve a further study [7].

Lechiguanas hantavirus (LECV) was initially described by Levis et al. [8] as a lineage associated with HPS cases in the central region of Argentina. This virus is mainly carried by the yellow pygmy rice rat (*Oligoryzomys flavescens*). Sporadic host-switch and/or spillover infections were found in other rodents such as the black-footed pygmy rice rat (*Oligoryzomys nigripes*) [9, 10]. HPS cases in Uruguay were first recognized in 1997. Studies on the rodent hosts and viral lineages performed between 1999 and 2005 showed that HPS cases in Uruguay were mainly in the southern region of the country and during the spring-summer seasons; LECV and the closely related Andes Central Plata hantaviruses were the viral lineages most frequently associated with HPS cases and the primary reservoir host in Uruguay for both viral lineages is the yellow pygmy rice rat [11, 12]. Since the first HPS case recognition in 1997, annual incidence varied yearly, with Montevideo, San José and Canelones departments accounting for most (64%) HPS cases. A gradual increase in the incidence rate trend was observed, specially after 2000, when case surveillance was already well established. Lethality ranged between 9 and 50%, with a polynomial trend of about 20% between 2001 and 2017 [13].

An increasing number of studies have confirmed the use of species distribution models as an effective tool in the prediction of diseases affecting human health [14, 15], further establishing a new scientific discipline called pathogeography [16] as an essential framework for the geographical analysis of zoonotic diseases affecting humans [17, 18]. Assessment and analysis of the potential distribution of rodent reservoir hosts and the influence of environmental factors on hantavirus transmission can be useful in understanding the spatial patterns of disease transmission risk [19, 20].

The present study aimed to update the information on the hantavirus reservoir hosts and viral lineages in Uruguay and model the potential distribution of yellow pygmy rice rats as relevant hantavirus hosts in a South American region.

## 2. Methods

### 2.1. Study Area and Rodent Sampling

Between 2008 and 2014, trapping expeditions were carried out in two different areas: likely sites of exposure for HPS cases and localities not associated with HPS cases (Table 1). The study included areas not previously examined for hantavirus infection in rodents as well as new localities from the southern area endemic for HPS.

**Figure 1:**
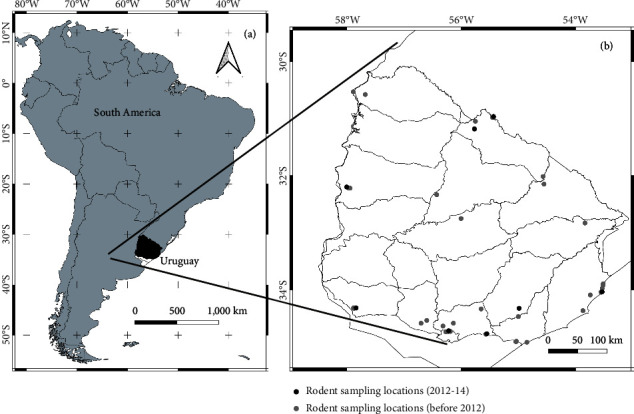
(a) Uruguay as a study area in the general context of South America. (b) Distribution of serological analysis points for *Oligoryzomys flavescens* rodents in Uruguay. Territories sampled for rodents between 2012 and 2014 are shown with black dots, and territories sampled before 2012 are shown with gray dots. The country layer was obtained from https://www.naturalearthdata.com and licensed CC BY. The maps were developed using QGIS (https://www.qgis.org) with the composer tool. The final composition was created using CorelDRAW 2018.

Sites associated with cases included the places where HPS patients had lived or worked in 6 weeks before the onset of symptoms and nearby natural habitats, including peridomestic areas, natural ecosystems, or agroecosystems (cultivated areas, shrublands, wetlands, road borders, brook borders, and natural and forestry with exotic species (*Eucalyptus* spp. and *Pinus* spp). Expeditions not linked to cases included those in natural ecosystems, small agroecosystems, and the mentioned forestry (Figure 1).

Small mammals were trapped with Sherman live capture traps (model LFATDG; 23 cm × 8 cm × 8.5 cm; Sherman Traps Inc., Tallahassee, FL) according to established bioethical and biosafety guidelines [21] and processed as previously described [12, 22]. Blood and organ samples (lung, kidney, liver, and spleen) were collected. Blood was obtained from the retro-orbital sinus and stored at 4°C, and tissues were maintained in liquid N_2_ until arrival at the laboratory and then stored at −80°C. Specimens were identified in the field by external characteristics and deposited in the specimen collection of the Sección Zoología Vertebrados in the Facultad de Ciencias, Universidad de la República, Montevideo, Uruguay with the following numbers: ZVC-M 5736 (1434), ZVC-M 5737 (1574), ZVC-M 5738 (1586), and ZVC-M 5739 (1626).

For the calculation of Shannon's diversity index, we used the Vegan package [23].

### 2.2. Anti-Hantavirus IgG ELISA and RT-PCR Genome Amplification

IgG antibodies to hantavirus were detected through an ELISA that used Maciel virus (MACV) antigen consisting in an inactivated, 3M rad gamma-ray irradiation detergent-extracted lysate of Vero-E6 infected cells, with a 100% infection index controlled by indirect immunofluorescence (kindly supplied by Dr. Silvana Levis, INEVH, Pergamino, Argentina). Polyvinyl chloride microtiter plates (Thermo Fisher Scientific Inc., Walthman, MA) were coated overnight at 4°C with the antigen, uninfected Vero-E6 cell culture antigen was used to determine the specificity of the mouse antibodies, both were used at a dilution 1 : 1000−1 : 2000, according to the supplier instructions. Unbound antigen was removed by washing three times with phosphate-buffered saline (PBS)-Tween 20, 0.1% (Sigma–Aldrich, St. Louis, MO). After blocking with PBS-Tween 20, 0.1%-dry milk 5% (37°C, 1 h), sera diluted fourfold, beginning with 1 : 100, were added. Bound antigen was measured by using a mix of goat anti-*Peromyscus leucopus* IgG (H + L) and goat anti-rat IgG (heavy- and light-chain-specific; Kirkegaard & Perry Laboratories, Gaithersburg, Maryland, USA) conjugated to horseradish peroxidase. A hyperimmune mouse ascitic fluid and an uninfected Balb-C mice serum were used as positive and negative controls, respectively. Optical densities (ODs) at 405 nm were measured on a microplate spectrophotometer (Labsystems Multiskan EX; Thermo Labsystems, Vartaa, Finland), and the ODs of the uninfected antigen-coated well were subtracted from that of its corresponding viral antigen to yield the adjusted OD. A serum was considered positive if OD was >0.2 U after adjustment (OD in the noninfected antigen was subtracted to the OD measured with the MAC-infected antigen). Sera were initially screened at a 1 : 100 dilution; positive sera were then subjected to titration and only sera with a titer >1 : 400 were considered positive.

Total RNA extraction from the lung tissues of seropositive rodents was carried out as follows: lung or liver tissues (100 mg) were treated with 1 mL of TRIzol® according to the manufacturer's instructions [22, 24]. Reverse transcriptase from Moloney murine leukemia virus (MMLV) (Thermo Fisher Scientific, Waltham, MA) or Superscript II (Invitrogen™, Thermo Fisher Scientific, Waltham, MA) were used for the reverse transcription. Briefly, 5 *µ*L of total RNA were mixed with 20 pmol of the (+) primer, 0.8 mM of each dNTP and 200 U reverse transcriptase in a final volume of 20 *µ*L. Nested or seminested RT-PCRs were performed to achieve phylogenetically informative *S* segment partial sequences [22, 24]. The first round of PCR was carried out with 5 *µ*L of first strand cDNA in a final volume of 50 *µ*L with 10 pmol of each specific primer and 0.3 *µ*L of Platinum Taq® DNA polymerase (Invitrogen™, Thermo Fisher Scientific, Waltham, MA). Second-round (nested or seminested PCR) was performed using 1 or 2 *µ*L of the first-round product. All reactions were performed in a PCR Sprint Thermal Cycler (Thermo Electron Corporation, USA) (detailed protocols are available in the supplementary information file; primer sequences are listed in Table S1).

### 2.3. Sequencing and Phylogenetic Analysis

PCR products were purified using a QIAquick gel extraction kit (QIAGEN Inc., Valencia, CA) and sequenced with the same primers used for the RT-PCRs at the MACROGEN® facility (South Korea). Sequences of 362 and 162 nucleotides of two partial *S* segment regions were obtained with the primers listed in Table S1, and then aligned with hantavirus sequences downloaded from GenBank. Two Old World hantavirus sequences were used as outgroup species (Hantaan L37904 and Seoul NC005216). The final alignment for the ML analysis comprised 1285 nucleotides of the S segment. Editing and alignment were done using BioEdit v7.0.9.0 and Muscle [25]. To estimate the most suitable model of nucleotide substitution, Modelgenerator v0.85 software was used [26]. Phylogenetic reconstruction was done under the maximum likelihood criterion using PhyML 3.0 software [27]. Statistical support of the nodes was assessed through the approximate likelihood-ratio test (aLRT).

### 2.4. Modeling the Potential Distribution of the Yellow Pygmy Rice Rat in Uruguay

**Figure 2:**
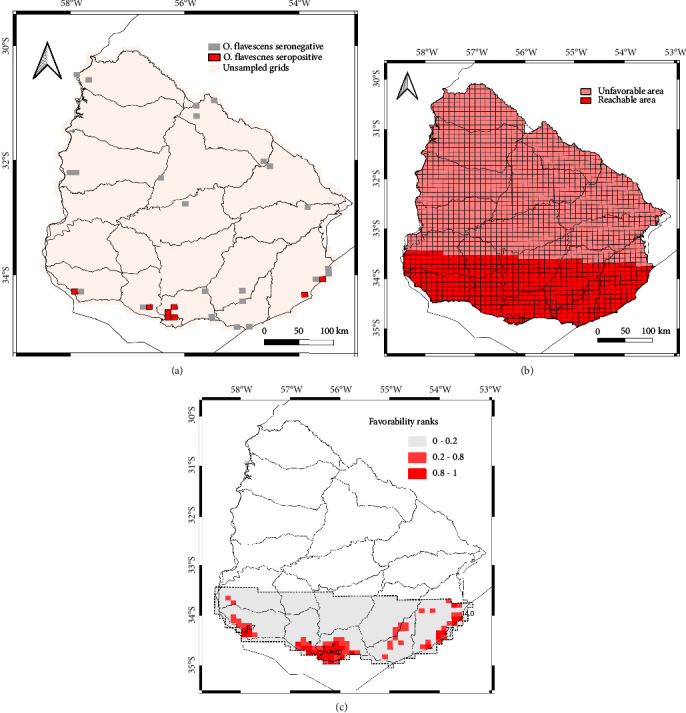
(a) Grids used to train the spatial model; grids sampled with hantavirus seronegative are indicated in gray, and grids with hantavirus seropositive (or hantavirus reservoir zone) in red. (b) Results of the spatial favorability model for O. *flavescens* as a hantavirus reservoir host, showing the area unfavorable or unreachable for the rodent as a hantavirus host (with spatial favorability values or *F* < 0.2) in pink and the reachable area (with spatial favorability values or *F* ≥ 0.2) in red. (c) Results of the environmental favorability model for O. *flavescens* as a hantavirus reservoir, showing the most favorable areas for the presence of a hantavirus reservoir or seropositive rodents (favorability values or *F* ≥ 0.8) in red, the intermediately favorable areas (0.2 ≤ *F* < 0.8) in pink, and the unfavorable areas (*F* < 0.2) in gray. The country layer was obtained from https://www.naturalearthdata.com and licensed CC BY. The grid layer was created with the tool “create grid” of the software QGIS https://www.qgis.org.

We revised the data collected from capture expeditions conducted by our research group from 1997–2007 (previous studies) and 2008–2014 (this work), together with data acquired from the Natural Museum of Natural History and the specimen collection of the Facultad de Ciencias, Universidad de la República, Uruguay. The yellow pygmy rice rats are the most prevalent reservoir hosts in the country; thus, we performed the modeling process using the data of forty-nine different geographical locations with serology information (Figures 1(b), 2(a), and Table S4). We applied a 10 × 10 km grid of Uruguay (1887 total cells) for the model procedure analysis using the QGIS program [28]. We modeled the *O. flavescens* distribution as a dependent variable based on a set of explanatory environmental variables, namely, spatial structure, topography, climate (temperature and precipitation), hydrology, land use, and other human activities, that could potentially affect it at the spatial resolution analyzed. We digitized different variables using QGIS tools (see variables and sources in Table S2).

Specifically, we applied the favorability function algorithm to infer the potential distribution of *O. flavescens* as the hantavirus reservoir in Uruguay and determine the environmental variables explaining the reservoir host distribution [29, 30]. In the modeling process, we used a factorial logistic regression. From the probability obtained from the logistic regression, we calculated the favorability function (FF) according to the following equation:

(1) $$\begin{array}{c} FF=\frac{\left[P/\left(1-P\right)\right]}{\left[\left(n1/n0\right)+\left(P/\left[1-P\right]\right)\right]}\text{,} \end{array}$$

where *P* is the probability value obtained according to each model and *n*1 and *n*0 are grid numbers corresponding to presence or absence (rodents testing seropositive or seronegative for hantavirus, respectively). We applied the FF to identify the areas favorable to the species [29] regardless of the presence/absence ratio. The FF reflects the degree (between 0 and 1) to which the probability values obtained in each model differ from those expected according to the species prevalence, where 0.5 indicates no difference between both probability values. We used the FF that combined the regression probability of occurrence of an event with the probability that such an event occurs at random [29, 30].

First, we built a spatial model to define the spatial structure of the territory of the seropositive *O. flavescens* rodents in Uruguay. We considered a polynomial trend-surface analysis that included a quadratic and cube effect of latitude and longitude and interactions between them (Lo, Lo2, Lo3, La, La2, La3, LaLo, La2Lo, and LaLo2) [31, 32]. In biogeography, spatial structure is a reflection of purely spatial trends derived from biological processes such as history, spatial ecology, dispersal events, and population dynamics [31, 33, 34]. For the entire country, we performed a logistic regression of seropositive rodent distribution on latitude (La), longitude (Lo), and the interactions described between them. Specifically, we performed a backward stepwise logistic regression with each event (*O. flavescens* hantavirus seropositive and seronegative individuals) and the nine spatial terms as predictor variables to remove the nonsignificant spatial terms from the models [32]. In the modeling procedure, we included the resulting linear combinations (ysp) as the spatial variable without nonsignificant spatial terms to produce the geographical distribution models. Then, we obtained a cartographic favorability model representing the values of spatial favorability or accessible territory for *Oligoryzomys* as a hantavirus reservoir. Spatial favorability values greater than 0.2 (*F* ≥ 0.2) are indicated as areas geographically accessible to hantavirus-infected yellow pygmy rice rats, and those less than 0.2 as areas not currently accessible to the hantavirus reservoir.

Then, from seropositive and nonseropositive rodents and the set of environmental variables grouped into environmental factors (topography, climate, environment, hydrology, land use, lithology, and other human activities), we ran the environmental favorability model only in the spatial territory accessible to the rodent viral host. To run the environmental model, we performed a multifactorial logistic regression from predictive explanatory variables and the presence/absence data (hantavirus seropositive and nonseropositive yellow pygmy rice rats) according to a forward-backward stepwise procedure based on Akaike information criteria (AIC). For this, we used the fuzzySim package [35] from R [36]. Finally, we estimated the relative weight of each variable included in the model using the Wald test parameter, which relates the coefficient of each variable according to its significance with the variation coefficient of that coefficient [37]. The Wald test was applied using the survey package [38, 39].

We represented spatial and environmental favorability models using QGIS (QGIS Development Team, 2020). For the environmental model, environmental favorability values were categorized into high (areas with favorability values equal to or higher than 0.8), intermediate (favorability values equal or higher than 0.20 and less than 0.80), and low (favorability values less than 0.20). This categorization is equivalent to defining a prediction with odds higher than 4 : 1 as favorable and lower than 1 : 4 as unfavorable [40].

Finally, we evaluated the discrimination capacity of the model with the area under the curve (AUC) of the receiving operating characteristic (ROC), which is independent of any favorability threshold [41, 42]. To evaluate the classification capacity of the models, we used the following five indices: sensitivity, specificity, underprediction rate (UPR), overprediction rate (OPR), and true skill statistic (TSS). The classification indexes used ranged from 0 to 1. The TSS index is a general measure of classification obtained by subtracting true positive from false positive rates and is independent of the prevalence. The TSS ranges from −1 to +1, where a TSS = 1 indicates perfect agreement, and values of TSS ≤0 indicate a performance no better than random [43]. The modEvA package was used to evaluate the performance of the model [44].

## 3. Results

### 3.1. Rodent Distribution by Species and Capture Site and Hantavirus Serology

During 7140 trapping nights, 430 micromammals were captured. Rodents belonged to the Muridae (subfamily Murinae), Cricetidae (subfamily Sigmodontinae), and Caviidae families. A marsupial from the Didelphidae family was also captured. Trapping efforts ranged from 320 to 920 trap/nights and trapping efficiencies varied from 0.43% to 15% (Table 2). Trapped rodents belonged to 13 species. The most abundant captures were Azara's grass mice (*Akodon azarae*), followed by specimens of the *Oxymycterus* sp., swamp rats (*Scapteromys tumidus*), yellow pygmy rice rats (*Oligoryzomys flavescens*), house mice (*Mus musculus*), and black-footed pygmy rice rats (*Oligoryzomys nigripes*).

Species diversity for each trapping site was assessed though Shannon's diversity index (Table 2), the highest value was found at San Pedro (Colonia) and the lowest corresponded to the Villa Serrana expedition.

Serum specimens collected from 430 rodents were screened for IgG antibodies to MACV by ELISA. Anti-MACV antibodies were detected in five rodents from four different locations (Table 3). Four yellow pygmy rice rats tested seropositive, three of them were captured during expeditions to Laguna de Castillos, Punta del Diablo, and Las Piedras. They were captured in natural habitats such as “ombú” (*Phytolacca dioica*) bushes or wetlands, as well as in agroecosystems such as a tomato plantation. The fourth seropositive *O. flavescens* specimen was captured in San Pedro (Colonia department), together with a seropositive Azara's grass mouse. Both were caught at road borders.

### 3.2. Genomic Identification and Phylogenetic Analysis

Partial S segment genome amplification and sequencing was achieved for all seropositive rodents (Table 3). Maximum likelihood phylogenetic analysis included viral sequences from New World hantavirus lineages, together with previously reported Uruguayan sequences. Central and North American viruses were the most divergent and appear basal to the South American lineages. Hantavirus species and lineages from South America grouped in a single clade with a 0.98 aLRT support, which in turn splits into two main clades. One of these clades grouped the Laguna Negra, Rio Mamoré-Maripa, Jaborá, and Alto Paraguay hantaviruses, with an aLRT support of 0.93, Choclo virus (originally identified in Panama) was part of this clade, but with a nonsignificant aLRT support. The second South American clade is in turn divided into the following two subclades: one included Juquitba and related lineages (as Araucaria and Itapúa) and the other clustered Andes, Lechiguanas (and related lineages as Bermejo and Ñeembucú), Hu39694, Maciel, Pergamino, Orán, Castelo dos Sonhos-Tunari, and the Araraquara-Paranoa lineages.

**Figure 3:**
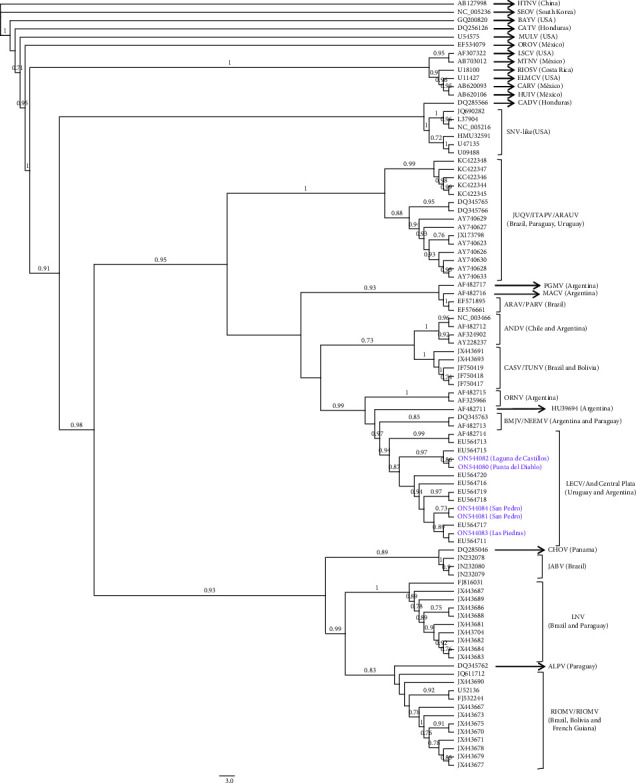
Maximum likelihood phylogenetic analysis: phylogeny was deduced using previously reported hantavirus and Uruguayan partial *S* segment sequences (rodents and HPS cases). The sequences reported here are colored in purple, and capture sites are indicated (GenBank accession numbers: ON544080, ON544081, ON544082, ON544083, and ON544084). Hantaan (AB127988) and Seoul (NC005236) sequences were used as the outgroup species. Approximate likelihood-ratio test (aLRT) was used to assess the statistical support of the clades, with only significant values shown. Abbreviations of hantavirus species and lineages: HTNV = Hantaan, SEOV = Seoul, BAYV = Bayou, CATV = Catacamas, MULV = Muleshoe, OROV = Rio de Oro, LSCV = Limestone Canyon, MTNV = Montano, RIOSV = Rio Segundo, ELMCV = El Moro Canyon, CARV = Carrizal, HUIV = Huitzilac, CADV = Caño Delgadito, SNV = Sin Nombre, JUQV = Juquitiba, ITAPV = Itapúa, ARAUV = Araucaria, PGMV = Pergamino, MACV = Maciel, ARAV = Araraquara, PARV = Paranoa, ANDV = Andes, CASV = Castelo Dos Sonhos, TUNV = Tunari, BMJV = Bermejo, NEEMV = Neembucú, LECV = Lechiguanas, CHOV = Choclo, JABV = Jaborá, LNV = Laguna Negra, ALPV = Alto Paraguay, RIOMV = Río Mamoré, and MARV = Maripa.

Hantaviral sequences from the yellow pygmy rice rats and from the only seropositive Azara's grass mouse clustered in a monophyletic group with sequences previously retrieved from yellow pygmy rice rats captured in Uruguay and Argentina. The group shared a nucleotidic and aminoacidic identity of 94.1% and 99.5%, respectively (Figure 3 and Table S3). All members belonged to the Lechiguanas (LEC)/Andes Central Plata lineages, which were initially described in specimens of *O. flavescens* from Uruguay and Central Argentina. The sequences reported herein belonged to the LEC/Andes Central Plata genotype but fell into two different subclades. Sequences from Laguna de Castillos, Punta de Diablo, and a 2004 sample from La Coronilla (Rocha, Eastern Uruguay) were more related; meanwhile, hantavirus from Southern and Western Uruguay (San Pedro in Colonia, Las Piedras in Canelones, and six previously reported sequences from the southern area) formed another subclade.

### 3.3. Potential Favorable Distribution of the Main Hantavirus Host in Uruguay

A favorability model for Uruguay was generated from the data on seropositive yellow pygmy rice rats (Figure 2(b)). This model established a spatial latitudinal boundary that limited the current reachable space for these rodents as hantavirus hosts to the southern half of Uruguay. Then, the environmental model only in that spatial area reachable by the seropositive rodents was obtained (Figure 2(c)). The most explanatory variables for the environmental model and their relative importance according to the Wald test are shown in Table 4. In summary, six variables were significant, belonging to the factors of land use, hydrology, climate, and environment. Territory transformed by human activity and the presence of high humidity were among the most explanatory variables. Regarding fit, the environmental model presented a high discrimination capacity (AUC > 0.9) or outstanding status according to Hosmer and Lemeshow [45] and a high capacity for the classification of presence (sensitivity values higher than 0.87), absence (specificity values higher than 0.92), and in general (TSS values higher than 0.8). Finally, the model had a UPR of less than 0.002 and an OPR of 0.86.

## 4. Discussion

Azara's grass mice, long-nosed mice, swamp rats, yellow pygmy rice rats, house mice, and black-footed pygmy rice rats accounted for 89% of the captures (Table 2). However, species distribution varied between sites. Azara's grass mice were the most captured rodents in San Pedro and Las Piedras, while long-nosed mice were the most captured rodents in Punta del Diablo, Cerro Chapeu, Los Titanes, and Tranqueras. House mice were the most trapped rodents only in Constancia (Paysandú). Yellow pygmy rice rats were captured from all sites and environments explored, although they were the most abundant only in Laguna de Castillos.

In San Pedro, an Azara's grass mouse tested seropositive, providing the first evidence of hantavirus infection in this rodent species in our country. Both seropositive rodents found in San Pedro were caught in human-disturbed habitats such as road borders. All samples showed high antibody titers; three of them had titers above 1 : 6400, while the San Pedro samples had a titer of 1 : 1600.

Except for the seropositive rodent from Laguna de Castillos, which was captured in a protected area ecosystem with low human disturbance, all other seropositive rodents were found in sites associated with human disturbance and/or agricultural activities, indicating a risk of infection for human inhabitants.

Captures from the northern part of the country (Tranqueras, Cerro Chapeu, and Constancia), whether associated with HPS cases or not, contained no seropositive individuals. The captures were carried out during months of high rodent activity (December–April) and the trapping effort was similar to or higher than that in other expeditions; however, trapping efficiencies were low, which may partially explain the lack of positive specimens. On the other hand, expeditions with similar or lower trapping efforts, such as those in San Pedro and Laguna de Castillos, resulted in higher trapping efficiencies and the collection of seropositive rodents. The expedition to Villa Serrana was undertaken in July at a probable site of infection for an HPS case but had very low efficiency despite the trapping effort. This could be due to low rodent activity because of the winter season and local population measures of rodent control after the HPS case.

Hantavirus seroprevalence varied greatly between sites. The lowest corresponded to the San Pedro capture where two seropositive species were found, as well as the highest species diversity recorded in the study with a Shannon index value of 1.8 (Table 2). Seroprevalence was 6.7% among yellow pygmy rice rats and 3.3% among Azara's grass mice. More in general, in this study, the proportion of positive rodents was the highest recorded in the country, above 6% and up to 17% (Las Piedras). The seropositive rodents were associated mainly with ecosystems affected by human activities but also with natural protected areas such as Laguna de Castillos. Our previous studies in the rural areas of Canelones, Montevideo, and San José (Southern Uruguay) showed prevalences up to 3% in *O. flavescens* [12]; additionally, the only previous record in La Coronilla (Rocha department) showed a seroprevalence of 1.7% [46].

The distribution and size of rodent populations and the proportion of hantavirus positive hosts is driven by multiple factors, including richness of the rodent species assembly, climate, geography, and anthropogenic disturbance of the ecosystem [47–49]. Yates et al. [50] showed a delayed rise in deer mouse (*Peromyscus maniculatus*) populations following “El Niño” episodes in Southwestern USA. This climatic condition led to a “trophic cascade”, wherein an increase in primary productivity provided more favorable conditions for rodent reproduction. Because prevalence of infection depends on many factors but may be roughly proportional to the rodent population density, authors propose that “El Niño” (ENSO) episodes influenced *P. maniculatus* populations, favoring conditions for the HPS outbreaks that occurred in 1993 and 1998 in Southwestern USA [50]. In Uruguay, 2012 and 2013 were ENSO phase and neutral years, respectively, but rainfall was above the expected level under both climatic phases (Figure S1). The increase in rodent seroprevalence in comparison with our previous records in the same areas could be associated with a rise in rodent population due to favorable environmental conditions. Similar findings were reported in Paraguay and Panama, where highly rainy years were associated with an increment in rodent populations and preceded HPS outbreaks [51].

It is worth noting that the proportion of positive yellow pygmy rice rats in 2013-2014 is not related to better trapping efficiency, conversely, capture efficiencies in this period were half those reported in the 2003 expeditions (6.02% vs. 12.8%) [12].

As described by several authors, habitat fragmentation due to agricultural activities and human disturbance may contribute to changes in rodent population assemblies, thus influencing hantavirus seroprevalence (reviewed in [51–53]). Overall, although the prevalence of infection in yellow pygmy rice rat populations may vary according to the environment and season, our records are comparable with the findings in the region [9, 54, 55].

Phylogenetic analysis showed a topology compatible with a similar S segment analysis reported previously, with Central and North American hantaviruses divergent from the South American lineages. The two main South American clades and the relationships of the sequences in the inner clades are well supported and consistent with other analyses performed on the nucleocapsid gene or the complete *S* segment [56–58], showing that there is sufficient phylogenetic information to assign our sequences to previously known species or lineages. The yellow pygmy rice rats and Azara's grass mouse sequences reported here clustered into the LEC/Andes Central Plata genotypes. In view of their high nucleotidic and aminoacidic identity and being a monophyletic group, these viruses should be considered Lechiguanas-like or LEC-like. In turn, the LEC-like sequences fall into two different subclades. Sequences from Laguna de Castillos, Punta de Diablo, and a 2004 sample from La Coronilla (all from Rocha department, Eastern Uruguay) are more related; meanwhile, hantavirus from Southern and Western Uruguay (San Pedro in Colonia, Las Piedras in Canelones, and six previously reported sequences from Southern Uruguay) form another subclade.

Azara's grass mouse was previously reported as the primary reservoir host of Pergamino virus (PGMV), a nonpathogenic South American hantavirus [8]. Our sequence analysis placed this sample in the LEC/Andes Central Plata lineage, closely related to the *O. flavescens* sample collected in the same locality (San Pedro, Colonia). In the San Pedro capture, Azara's grass mice were the most abundant rodents; meanwhile yellow pygmy rice rats were the fourth most abundant. Considering that the rodents were captured in the same trapping expedition and same transect, this finding may indicate a spillover infection from the reservoir host to Azara's grass mouse. The captures in this locality displayed the highest record of species richness (Table 2), together with the lowest seroprevalence for each rodent species. We have not specifically tested the relationship between host species diversity and hantavirus seroprevalence, since our sampling was not designed to achieve this objective. However, our results may suggest a “dilution effect,” a phenomenon in which species richness tends to diminish the seroprevalence in the rodent hosts, whereas a lower species richness in a given rodent assembly results in a higher proportion of infected rodents [59, 60, 61].

On the other hand, recent studies proposed that species identity and assemblage composition, instead of having a dilution effect, are likely the strongest forces driving hantavirus persistence in the rodent population [48]. A recent field study from Camp et al. [62] showed that augmenting food resources may change the composition of rodent communities, but it does not affect hantavirus seroprevalence over time and no dilution effect was observed, thus adding to the hypothesis that habitat composition is a primary driver in hantavirus prevalence. Importantly, the virus-host-environment ecology for a given virus is not generalizable, showing the relevance of local studies for risk assessment and epidemiology [62].

Several authors showed that changes in land use, from natural to anthropized, alter the landscape for rodent populations, destroying burrows and reducing the availability of shelters and food (insects), also affecting the predators that regulate the populations of the more generalist species. These factors cause a diversity loss in rodent communities in the short-medium term [63, 64]. Also, in certain ecosystems, a rise in rainfall increases the availability of food, thus favoring the populational growth of the more generalist rodents, reducing also their diversity [65, 66]. In this way, our model detected that the most explanatory variables for the seropositive rodents in Uruguay are the anthropic activity combined with rainfall local conditions [48]. Several studies on South American hantavirus have challenged the “one rodent species-one viral lineage” paradigm, demonstrating that a single viral lineage may infect different rodent species, either in the same or different geographical areas [10, 22, 67]. This supports the hypothesis that the community structure of sympatric host species is an essential contributor to *Orthohantavirus* dynamics.

The current distribution of *Oligoryzomys flavescens* [68] in South America encompasses Argentina, Brazil, Paraguay, and Uruguay territories [69–71]. Our previous studies indicate that the yellow pygmy rice rat is the main reservoir host for hantavirus in Uruguay [6, 22], and, like other generalist rodents, does not require specific environmental conditions for survival [70, 71]. However, hantavirus-infected yellow pygmy rice rats and the vast majority of HPS cases have been recorded in the southern half of the country, along with the infected Azara's grass mouse reported in this work. The distribution model trained from the positive rodents detected that their favorable environmental conditions are related to the presence of crops along with high humidity (local rainfall combined with coast proximity). This is in agreement with field studies from Brazil, USA, Panama and Paraguay indicating that anthropogenic landscape changes (habitat fragmentation, agriculture land use and forest loss) are strongly related with an increase in abundance in rodent hosts, followed by a rise in hantavirus seroprevalence [52, 72–75]. The incidence of climate conditions as temperature, rainfall and/or humidity in rodent abundances and seroprevalence is different if analyzed in temperate-arid, temperate or tropical environments. For example, Andreo et al. found a negative association with temperature together with a positive association with precipitation for a model combining *O. longicaudatus* presence and HPS cases in Argentina. In turn, Loehman et al. did not found a relationship between weather variables and rodent abundance in a temperate-arid region of USA [20, 76]. Our climate is classified as temperate-moderate-rainy with a temperature of the warmest month above 22° (Cfa in the Koppen classification) [77], different from the above mentioned studies, however it is reasonable to expect that a higher humidity may favor viral persistence in the environment, giving more chances of infection between hosts.

In summary, we explored new localities in Northern and Southern Uruguay associated with natural and artificial ecosystems. As result, we found that the yellow pygmy rice rat is still the most important hantavirus reservoir host in Uruguay. Although the yellow pygmy rice rat is also present in Northern Uruguay, we found no seropositive rodents in our expeditions to Cerro Chapeu and Constancia. Additionally, in Southern and Eastern Uruguay, we found hantavirus seroprevalences up to six times higher than those found in our previous studies. As corroborated by the environmental favorable model developed from seropositive yellow pygmy rice rats, their distribution may be explained by a combination of multiple factors, including more favorable climatic conditions of high humidity for rodent reproduction and virus survival in the environment, and increasing habitat fragmentation due to human activities such as agriculture and urbanization. Therefore, management tools for yellow pygmy rice rats and surveillance of Azara's grass mice as a potential virus source should be implemented in the favorable areas in the south of the country.

## 5. Conclusions

The integration of viral detection, genomic analyses, and the favorability function has proved to be a useful methodology to determine spatially accessible and environmentally favorable territories for the main hantavirus reservoir in Uruguay. The joint application of these innovative methodologies made it possible to quantitatively assess the potential risk territory for hantavirus infection in humans. We propose this integrative approach as a powerful tool that may be applied for the risk assessment of any vector-borne or zoonotic emerging disease in any territory.

## Figures and Tables

**Table 1:** List of localities sampled from 2008 to 2014 and their association with hantavirus pulmonary syndrome (HPS) cases.

Locality	Coordinates	Department	Year	Associated with HPS case
Laguna de Castillos	34°21′S/53°51′W	Rocha	Oct 2008	No
Punta del Diablo	34°2′S/53°32′W	Rocha	Mar 2012	Yes
Villa Serrana	34°19′S, 54°59′W	Lavalleja	Jul 2012	Yes
Cerro Chapeu	30°58′S/55°26′W	Rivera	Nov-Dec 2012	No
San Pedro	34°19′S/57°50′W	Colonia	Apr 2013	Yes
Las Piedras	34°43′S/56°13′W	Canelones	Dec 2013	Yes
Los Titanes-Guazuvirá	34°46′S/55°33′W	Canelones	Dec 2013	No
Constancia	32°12′S/58°00′W	Paysandú	Mar 2014	Yes
Tranqueras	32°11′S/55°46′W	Rivera	Dec 2014	No

**Table 2:** Rodents captured in the different trapping expeditions.

Locality, department ⟶	Laguna de Castillos	Punta del Diablo	Villa Serrana	Cerro Chapeu	San Pedro	Las Piedras	Los Titanes-Guazuvirá	Constancia	Tranqueras	Total per species	%
Species↓	Rocha	Rocha	Lavalleja	Rivera	Colonia	Canelones	Canelones	Paysandú	Rivera		
*Akodon azarae*	2	2	0	5	30	30	3	8	11	91	21.2
*Oxymycterus* spp.	1	10	0	8	25	0	21	0	13	78	18.1
*Scapteromys tumidus*	3	0	0	0	22	16	20	3	1	65	15.1
*Oligoryzomys flavescens*	13	7	2	2	15	6	3	2	9	59	13.7
*Mus musculus*	0	0	0	0	7	21	0	22	0	50	11.6
*Oligoryzomys nigripes*	11	0	1	1	26	0	0	0	0	39	9.1
*Necromys obscurus*	0	0	0	0	10	10	0	0	0	20	4.7
*Holochilus brasiliensis*	1	0	1	0	0	0	4	0	3	9	2.1
*Deltamys kempi*	3	2	0	1	0	0	0	0	1	7	1.6
*Rattus rattus*	0	4	0	0	1	0	1	0	0	6	1.4
*Cavia aperea*	0	0	0	0	0	0	3	0	0	3	0.7
*Calomys laucha*	0	0	0	0	0	0	0	1	0	1	0.2
*Reihtrodon* spp.	0	0	0	0	0	0	0	1	0	1	0.2
*Monodelphys dimidiata*	0	0	0	0	0	0	0	0	1	1	0.2
Total per locality	34	25	4	17	136	83	55	37	39	430	
*n* of species (species richness)	7	5	3	5	8	5	7	6	7		
Traps (night)	320	900	850	650	900	920	900	900	800		
Shannon's index	1.535	1.420	0.895	1.299	1.880	1.478	1.475	1.197	1.541		
Trapping efficiency (%)	10.6	2.8	0.47	2.6	15.1	9.0	6.1	4.1	4.9		

Trapping efficiency and species diversity (Shannon's index) are indicated.

**Table 3:** Hantavirus seroprevalence in wild rodents.

Locality/Department	Species	Sex	Lab number	Habitat	Antibody titer	Seroprevalence (%)	*S*segment amplification	GenBank accession number
Laguna de Castillos/Rocha	*O. flavescens*	Male	009	*Phytolacca dioica* (“ombú”) bushes	>1 : 6400	7.7	Yes	ON544082
Punta del Diablo/Rocha	*O. flavescens*	Female	1434	Wetland	>1 : 6400	14.0	Yes	ON544080
San Pedro/Colonia	*O. flavescens*	Male	1574	Road border	1 : 1600	6.7	Yes	ON544081
San Pedro/Colonia	*A. azarae*	Female	1586	Road border	1 : 1600	3.3	Yes	ON544084
Las Piedras/Canelones	*O. flavescens*	Male	1626	Tomato plantation	>1 : 6400	17.0	Yes	ON544083

Species, sex, and habitat where the rodents were captured, and antibody titer are shown. All seropositive samples were RT-PCR-positive and further sequenced. GenBank accession numbers are provided.

**Table 4:** Modeling the potential distribution of the yellow pygmy rice rat in Uruguay.

Environmental factor	Variables in the models	Wald
Land use	Crops (+)^1^	9.454475
Hydrology	Dist river (+)^2^	9.198434
Land use	Wetland (+)^3^	8.688036

Climatic	Max prep. (+)^4^	6.905434
	Annual prep. (BIO_12_) (−)^5^	6.257022

Environment	Dist cost (−)^6^	3.880827

^*∗*^Description of the input variables and units: 1, crops (%); 2, length of rivers (km); 3, wetland (%); 4, maximum average precipitation in 24 h (mm); 5, annual precipitation (mm); 6, distance to coast-continentality (km). Predictor variables included in the seropositive *O. flavescens* favorability environmental model ordered by decreasing relative importance according to Wald test values. The Wald parameter indicates the relative weight of every variable in each model. Signs in brackets show the positive or negative relationship between favorabilities and explicative variables in the models.

## Data Availability

The sequences reported in this study were submitted to GenBank under accession numbers ON544080, ON544081, ON544082, ON544083, and ON544084.

## Supplementary Materials

Supplementary Materials The supplementary file contains the RT-PCR protocols and primers used in the study (Table S1), the variables and factors used to build the distribution model of *O. flavescens* (Table S2), the genetic p-distances and identity percentages for the main South American hantavirus clades of the phylogenetic tree (Table S3), and the *O. flavescens* records used to build the distribution model (Table S4). The comparison of seasonal average rainfall during El Niño, Neutral and La Niña phases for the capture expedition localities is depicted in Figure S1.
