# Supplementary material for: Deciphering the Hantavirus Host Range Combining Virology and Species Distribution Models with an Emphasis on the Yellow Pygmy Rice Rat (Oligoryzomys flavescens)
Source: Transbound Emerg Dis. 2023 Apr 19;2023:2730050. doi: 10.1155/2023/2730050 (PMC12017062; doi:10.1155/2023/2730050)
Supplement: Supplementary Materials — The supplementary file contains the RT-PCR protocols and primers used in the study (Table S1), the variables and factors used to build the distribution model of O. flavescens (Table S2), the genetic p-distances and identity percentages for the main South American hantavirus clades of the phylogenetic tree (Table S3), and the O. flavescens records used to build the distribution model (Table S4). The comparison of seasonal average rainfall during El Niño, Neutral and La Niña phases for the capture expedition localities is depicted in Figure S1. [file 2730050.f1.docx]

**SUPPLEMENTARY INFORMATION**

**RT-PCR protocols used in the study**

| Buffer First strand 5x | 4uL |
| --- | --- |
| Hancu 1+ or Han 1(+) (10mM) | 2uL |
| DTT 0.1M | 2uL |
| dNTPs mix (10mM) | 1uL |
| Reverse transcriptase (200u/uL) | 1uL |
| H_2_O (to 15uL) | 5uL |
| RNA | 5uL |

Reverse transcription

45°C/60 min.

70°C/15 min.

1º Round PCR

| Buffer PCR 10X | 5uL |
| --- | --- |
| MgCl_2_ 50mM | 1.5uL |
| dNTPs mix (10mM) | 1uL |
| Hancu 1+ (10mM) | 4uL |
| Hancu 1 – (10mM) | 4uL |
| Taq pol (5u/uL) | 0.3uL |
| H_2_O (to 45uL) | 33.2uL |
| cDNA | 5uL |

Cycles:

94 ºC / 2 min.

94 ºC / 30”

55 ºC / 1 min. 40x

72 ºC / 30”

72ºC / 5 min.

2º Round PCR

| Buffer PCR 10X | 5uL |
| --- | --- |
| MgCl_2_ 50mM | 1.5uL |
| dNTPs mix (10mM) | 1uL |
| Hancu 2+ (10mM) | 4uL |
| Hancu 2 – (10mM) | 4uL |
| Taq pol. (5u/uL) | 0.3uL |
| H_2_O (to 49uL) | 33.2uL |
| 1° round | 1uL |

Cycles:

94 ºC / 2 min.

94 ºC / 30”

50 ºC / 2 min. 40x

72 ºC / 30”

72 ºC / 5 min.

| Buffer PCR 10X | 5uL |
| --- | --- |
| MgCl_2_ 50mM | 0.75uL |
| dNTPs mix (10mM) | 1uL |
| Han 1+ (10mM) | 1uL |
| Han 28 – (10mM) | 1uL |
| Taq pol (5u/uL) | 0.7uL |
| H_2_O (to 45uL) | 33.2uL |
| cDNA | 5uL |

1º Round PCR

Cycles:

94°C/ 3 min.

94°C/ 30 ´´

48°C/30´´ 40x

72°C/ 1 min.

72°C/5 min.

| Buffer PCR 10X | 5uL |
| --- | --- |
| MgCl_2_ 50mM | 0.75uL |
| dNTPs mix (10mM) | 1uL |
| Han 2+ (10mM) | 1uL |
| Han 11 – (10mM) | 1uL |
| Taq pol (5u/uL) | 0.7uL |
| H_2_O (to 48uL) | 33.2uL |
| 1° round | 1uL |

2º Round PCR

Cycles

94°C/ 3 min.

94°C/ 30 ´´

52°C/ 30´´ 40 x

72°C/ 1 min.

72°C/ 5 min.

**Table S1.-** Primers used in this study.

| **Primer name** | **Sequence** | **Position  (S segment)** | **PCR** | **Expected size  (bp)** | **Citation** |
| --- | --- | --- | --- | --- | --- |
| Han1 (+) | AGCACATTACAAAGCAGACGGCA | 166-189 | primary | 888 | Raboni et al (2005), Delfraro et al (2008) |
| Han28 (-) | AGCCATGATTGTGTTGCG | 1071-1054 | primary |  |  |
| Han2 (+) | CCAGTTGATCCAACAGGG | 274-291 | nested | 416 |  |
| Han11 (-) | TATGATATTCCTTGCCTTCACTTGGGC | 690-664 | nested |  |  |
|  |  |  |  |  |  |
| HanCu1 (+) | TGGGTITTYKCWKGIGCICCWGA | 958-984 | primary | 332 | Tenorio, A. (ISCIII, Spain, unpublished) |
| HanCu1 (-) | TTIRIGGYTGRTTIGAIATYTC | 1324-1301 | primary |  |  |
| HanCu2 (+) | CAGGAYATGMGRAAYACMATIATGGC | 1052-1073 | nested | 183 |  |
| HanCu2 (-) | TCIGGRTCCATRTCRTCICC | 1257-1237 | nested |  |  |

**Table S2.** Variables and factors that were used to build the distribution model of *O. flavescens*.

| CODE | VARIABLES | CODE | VARIABLES |
| --- | --- | --- | --- |
| Spatial | | | |
| YSp | Spatial logit^1^ (linear polynomial combination of Latitude (ºS) and Longitude (ºW) from the spatial logistic regression) | | |
| Topography | | | |
| A | Average altitude (m) ^(2)^ | S | Slope (◦) (calculated from altitude) |
| Ori-NS | Orientation; degrees of exposure North-South (calculated from slope) | Ori-EW | Orientation; degrees of exposure East-West (calculated from slope) |
| Rough | Roughness (m) |  |  |
| Climatic | | | |
| BIO_1_ | Average annual temperature (ºC) ^(3)^ | BIO_11_ | Mean annual temperatures of the coldest quarter (ºC) ^(3)^ |
| BIO_2_ | Mean diurnal range temperatures (ºC) (ºC) ^(3)^ | BIO_12_ | Annual precipitation (mm) ^(3)^ |
| BIO_3_ | Isothermality (BIO_2_/BIO_17_) (*100) (ºC) ^(3)^ | BIO_13_ | Precipitation of the wettest month (mm) ^(3)^ |
| BIO_4_ | Seasonal temperatures (ºC) ^(3)^ | BIO_14_ | Precipitation in the driest month (mm) ^(3)^ |
| BIO_5_ | Maximum temperatures in the warmest month (ºC) ^(3)^ | BIO_15_ | Seasonal precipitation (mm) ^(3)^ |
| BIO_6_ | Minimum temperatures in the coldest month (ºC) ^(3)^ | BIO_16_ | Precipitation in the wettest quarter (mm) ^(3)^ |
| BIO_7_ | Annual temperature range (BIO_5_-BIO_6_) ^(3)^ | BIO_17_ | Precipitation in dry quarter ^(3)^ |
| BIO_8_ | Mean annual temperatures of the wettest quarter ^(3)^ | BIO_18_ | Precipitation in the warmest quarter ^(3)^ |
| BIO_9_ | Mean annual temperatures in the dry quarter ^(3)^ | BIO_19_ | Precipitation in coldest quarter ^(3)^ |
| BIO_10_ | Mean annual temperatures in the warmest quarter ^(3)^ | PMax | Maximum average precipitation in 24h (mm) ^(3)^ |
| ETP | Potential evapotranspiration (mm) *^(3)^* | ETR | Monthly real evapotraspiration (mm) ^(3)^ |
| WatBalAut | Water balance in Autumn (mm) ^(3)^ | WatBalSpring | Spring water balance (mm) ^(3)^ |
| WatBalWint | Water balance in Winter (mm) ^(3)^ | WatBalSumm | Water balance in Summer (mm) ^(3)^ |
| BhAnn | Annual water balance (mm) ^(3)^ | AnnHum | Annual average humidity (mm) ^(3)^ |
| ColdQuaHum | Coldest quarter average humidity (mm) ^(3)^ | WarmQuaHum | Warmest quarter average humidity (mm) ^(3)^ |
| AnnHum | Annual average humidity (mm) ^(3)^ | Frost | Average number of frost days ^(3)^ |
| Other | | | |
| NDVI | Index of greenness (plant biomass productivity indicator) ^(4)^ | DistCost | Distance to coast- continentality (km) ^(5)^ |
| SunRad | Sun radiation (kwh/m2/day) ^(6)^ |  |  |
| Hydrology | | | |
| DistRiver | Minimum distance to rivers (km) ^(7)^ | LengRiver | Length of rivers (km) ^(7)^ |
| Land use | | | |
| Forests | Natural forests (%) ^(8)^ | Reforested | Reforestation (%) ^(8)^ |
| NatField | Natural field (%) ^(8)^ | Crops | Crops (%) ^(8)^ |
| Wetland | Wetland (%) ^(8)^ |  |  |
| Lithology | | | |
| SoilDepth | Soil depth ^(9)^ | TextSoil | Texture soil ^(9)^ |
| SoilRocky | Soil rocky ^(9)^ | FloodSoil | Flooded soil ^(9)^ |
| Human activities | | | |
| PopDen | Population density ^(10)^ | UrbGro | Urban ground (%) ^(10)^ |
| DistUrban | Minimum distance to the main urban centers (km) ^(11)^ | SumRoads | Lenght of roads and unpaved routes (m) ^(11)^ |
| DistRoad | Minimum distance to paved roads (km) ^(11)^ | DistUnpavRoad | Distance to unpaved roads (km) ^(11)^ |

Sources:

(1) Spatial variables, latitude and longitude, were generated from the QGIS (www.qgis.org) program according to the vector geometry tools: a) with "centroids of polygons", the centroid of each cell was calculated, and b) with "Export / Add columns of geometry", the values of length and latitude expressed in the 1984 World Geodetic System were allocated to each centroid (WGS84).

(2) United States Geological Survey 1996. GTOPO30. Land Processes Distributed Active Archive Center. – EROS Data Center, https://lta.cr.usgs.gov/GTOPO30. (accessed in April 2016).

(3) Ceroni (2008) from DNM-INIA. Monthly data series of thirty years for Uruguay (from 1980 to 2009). The operations to calculate the bioclimatic variables (BIO1–BIO19) were based on WorldClim. Global Climate Data available in Fick & Hijmans (2017).

(4) [https://www.vito-eodata.be](https://www.vito-eodata.be/), from SPOT-VEGETATION – S10 NDVI.

(5) It was generated with the QGIS (www.qgis.org) program calculating the average distance from the centroid of the grid to the coastline layer.

(6) Mapa Solar del Uruguay, versión 1.0, Memoria Técnica. G. Abal, M. D'angelo, J. Cataldo y A. Gutiérrez. Facultad de Ingeniería, Universidad de la República. June 4, 2010. <https://www.fing.edu.uy/if/solar/memoria-mapa-solar-v1.pdf> _(accessed in October 2010)_.

(7) It was generated with the QGIS (www.qgis.org) program, calculating the average distance from the centroid of the grid to the river line layer.

(8) Oficina de Planeamiento y Presupuesto (accessed in March 2010).

(9) Panario, D. & Gutiérrez, O. (2011). Mapa de ambientes: Cartografía implementada en un SIG. In: Mapa de Ambientes de Uruguay y Distribución potencial de especies, Convenio MGAP/PPR-CIEDUR, Montevideo.

(10) Instituto Nacional de Estadística (accessed in June 2011).

(11) It was generated from topographic charts 1.50,000 digitized by the Ministerio de Transporte y Obras Públicas.

**Table S3.-** p-distances and identity percentages (nucleotidic and aminoacidic) for the main South American hantavirus clades analyzed in the Figure 2 phylogenetic tree.

|  | **p-distance (Intragroup)** | **Identity%** | **p-distance (Intragroup )** | **Identity%** |
| --- | --- | --- | --- | --- |
|  | **nucleotides** | | **aminoacids** | |
| **LEC/Andes Central Plata** | 0.059 | 94.1 | 0.005 | 99.5 |
| **AND** | 0.047 | 95.3 | 0.003 | 99.7 |
| **ORN** | 0.006 | 99.4 | 0.002 | 99.8 |
| **JUQ** | 0.066 | 93.4 | 0.006 | 99.4 |
| **ARA** | 0.089 | 91.1 | 0.012 | 98.8 |
| **BMJ** | 0.07 | 93.0 | 0.007 | 99.3 |
| **LNV** | 0.008 | 99.2 | 0.001 | 99.9 |
| **MAC** | 0 | 100 | 0 | 100 |
| **RIOM** | 0.077 | 92.3 | 0.015 | 98.5 |
| **PGM** | 0 | 100 | 0 | 100 |

Table S4: *O. flavescens* records used to build the distribution model. Seropositive rodents are indicated in red.

| **Latitude** | **Longitude** | **Localities** |
| --- | --- | --- |
| -34,907 | -55,042 | Punta Ballena |
| -34,533 | -56,603 | Punta Valdez |
| -34,333 | -55,653 | Piedritas |
| -34,633 | -56,317 | Cerrillos |
| -34,733 | -56,267 | Melilla |
| -34,583 | -56,133 | Sauce |
| -34,362 | -53,870 | Laguna de Castillos |
| -34,364 | -53,869 | Laguna de Castillos |
| -33,974 | -53,531 | Santa Teresa |
| -33,891 | -53,514 | La Coronilla |
| -32,336 | -56,426 | Rincón del Bonete |
| -32,829 | -53,834 | Rincón de Ramírez |
| -30,581 | -57,675 | Colonia Palma |
| -34,038 | -53,548 | Punta de Diablo |
| -34,040 | -53,551 | Punta de Diablo |
| -34,040 | -53,551 | Punta de Diablo |
| -34,040 | -53,551 | Punta de Diablo |
| -34,036 | -53,552 | Punta de Diablo |
| -34,036 | -53,551 | Punta de Diablo |
| -34,322 | -54,988 | Villa Serrana |
| -34,322 | -54,988 | Villa Serrana |
| -30,982 | -55,433 | Cofusa |
| -30,984 | -55,447 | Cofusa |
| -34,318 | -57,877 | San Pedro |
| -34,317 | -57,878 | San Pedro |
| -34,318 | -57,876 | San Pedro |
| -34,321 | -57,868 | San Pedro |
| -34,318 | -57,876 | San Pedro |
| -34,320 | -57,878 | San Pedro |
| -34,317 | -57,876 | San Pedro |
| -34,313 | -57,878 | San Pedro |
| -34,317 | -57,876 | San Pedro |
| -34,316 | -57,877 | San Pedro |
| -34,087 | -53,741 | Laguna Negra |
| -34,467 | -55,000 | Sierra Carapé |
| -34,776 | -55,565 | Los Titanes |
| -34,776 | -55,565 | Los Titanes |
| -34,727 | -56,192 | Las Piedras |
| -34,728 | -56,190 | Las Piedras |
| -34,727 | -56,187 | Las Piedras |
| -32,213 | -57,976 | Constancia |
| -32,220 | -57,940 | Constancia |
| -34,917 | -54,852 | Maldonado |
| -34,906 | -55,033 | Punta Ballena |
| -34,583 | -56,703 | Punta Valdez |
| -34,633 | -56,317 | Los Cerrillos |
| -34,733 | -56,267 | Melilla |
| -34,583 | -56,133 | Los Cerrillos |
| -33,917 | -53,517 | La Coronilla |
| -32,750 | -56,255 | Rincón del Bonete |
| -32,150 | -54,552 | Cerro Largo |
| -32,017 | -54,567 | Cerro Largo |
| -30,983 | -55,455 | Cofusa |
| -31,050 | -55,755 | Cofusa |
| -30,533 | -57,883 | Bella Unión |

**Figure S1.-** Comparison of seasonal average rainfall during El Niño, Neutral and La Niña phases for the localities where capture expeditions were done. Data from past capture expeditions done between 2003-2006 are compared with the results presented in this work. The graphics were generated from the site: <http://iridl.ldeo.columbia.edu/maproom/ENSO/Impacts.html>
